# Supplementary material for: Flaxseed Powder Attenuates Non-Alcoholic Steatohepatitis via Modulation of Gut Microbiota and Bile Acid Metabolism through Gut–Liver Axis
Source: Int J Mol Sci. 2021 Oct 8;22(19):10858. doi: 10.3390/ijms221910858 (PMC8509295; doi:10.3390/ijms221910858)
Supplement: Supplementary file 1 [file ijms-22-10858-s001.zip › Supplementary Figures.pdf]

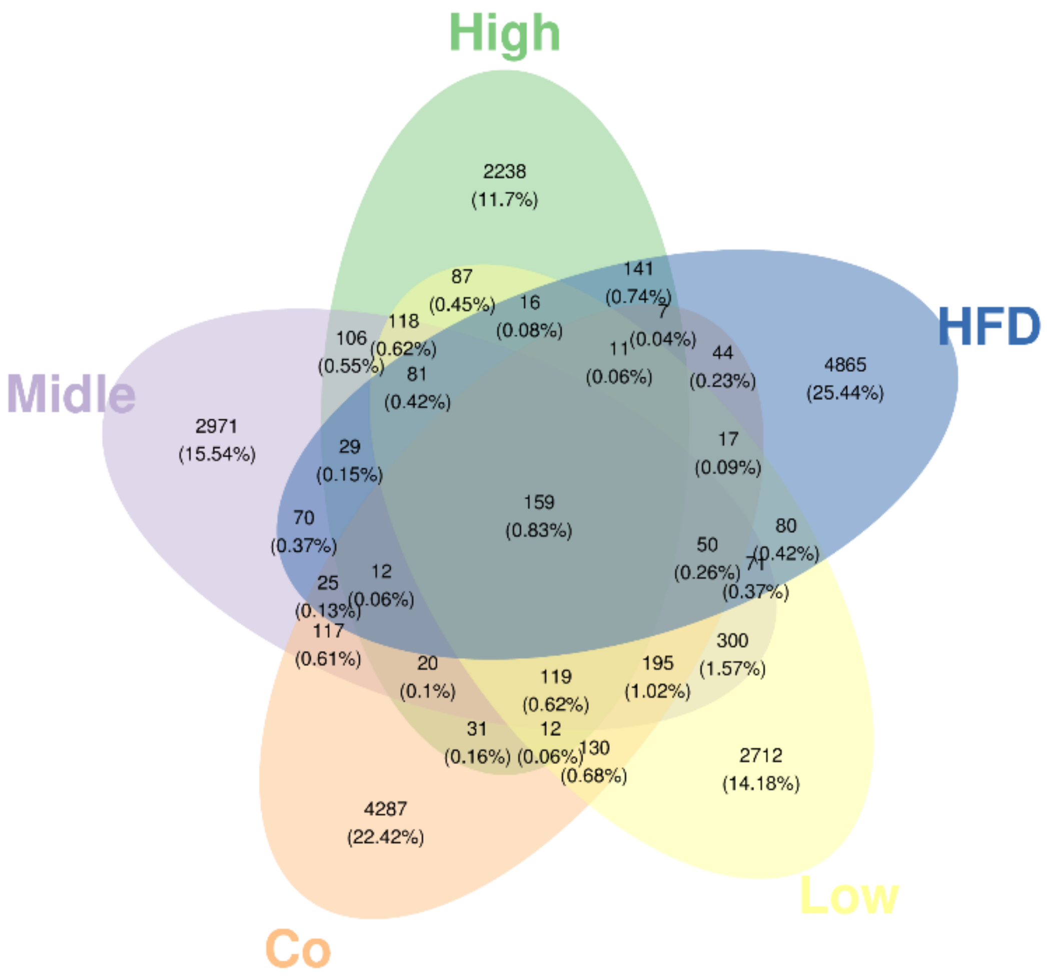

Figure S1. the OTU Venn diagram; Co, control group; HFD, high-fat diet; Low, 10% flaxseed group; Middle, 20% flaxseed group, High, 30% flaxseed group.

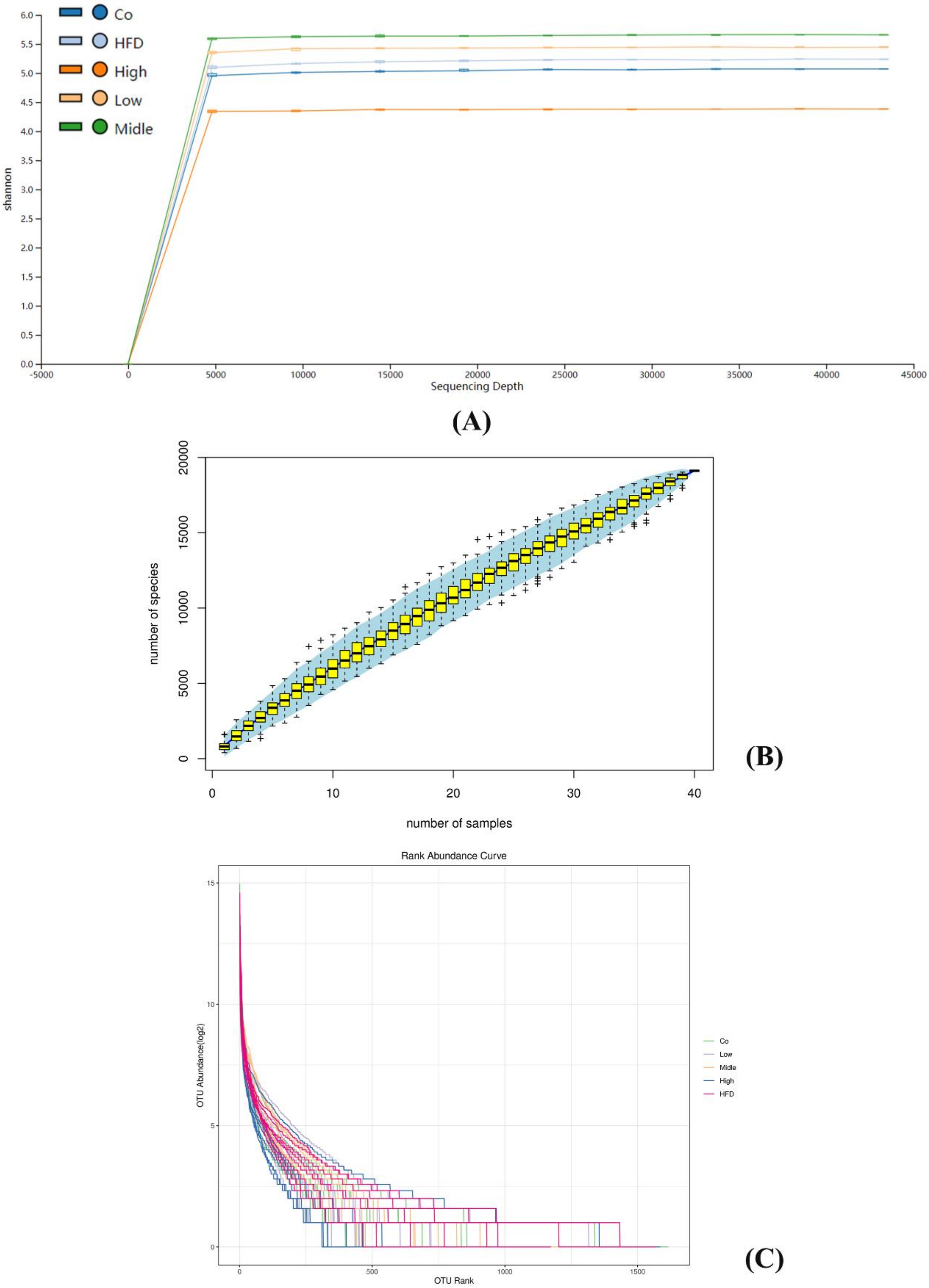

Figure S2 Rarefaction curve (A); Shannon index curve (B) and rank abundance curve (C). Co, control group; HFD, high-fat diet; Low, 10% flaxseed group; Middle, 20% flaxseed group, High, 30% flaxseed group.

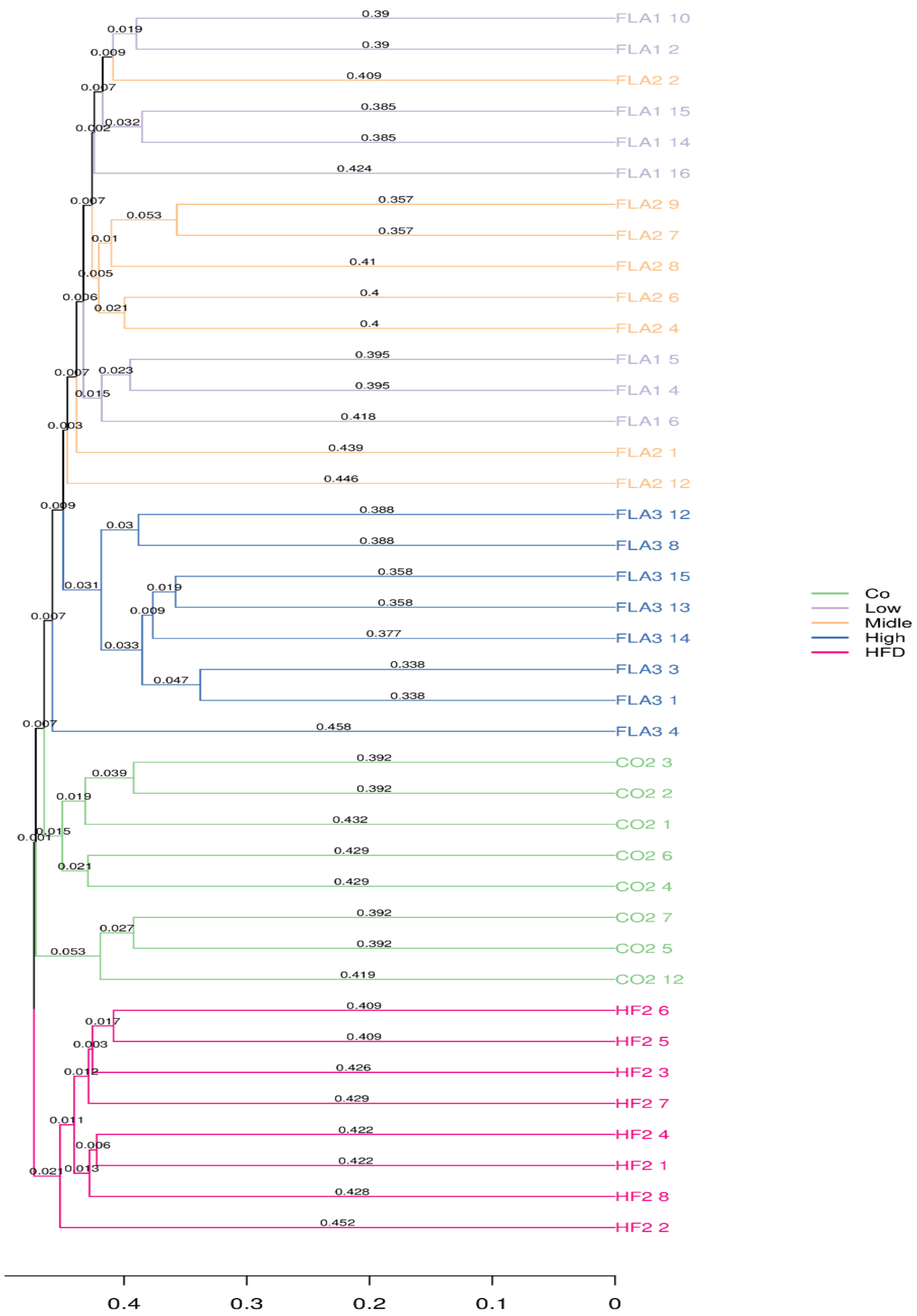

Figure S3. Hierarchical clustering based on the Jaccard similarity of fecal content microbial composition in control (n = 8), three LFA-fed (n = 8 each group) and HFD (n = 8) groups.

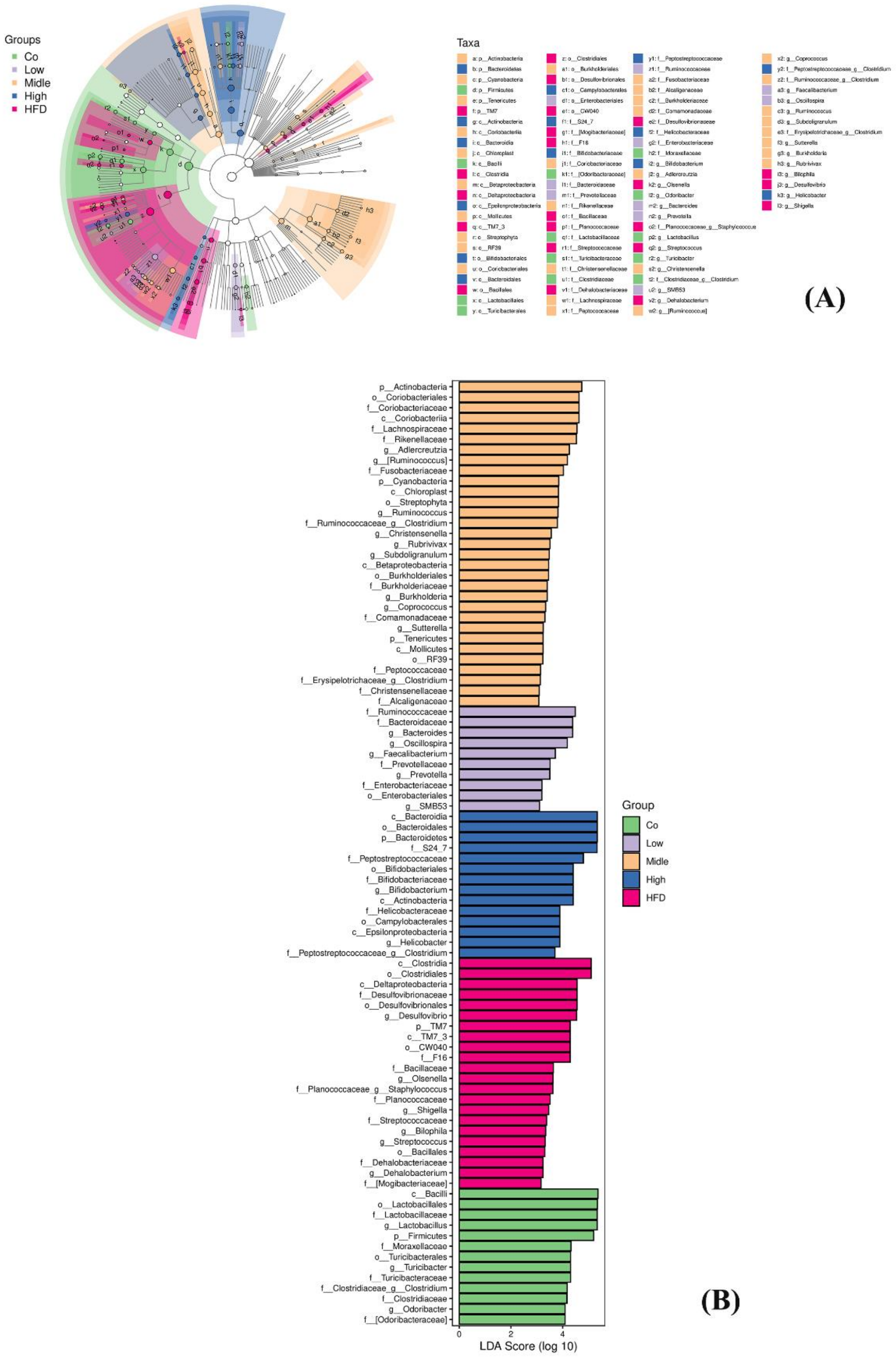

Figure S4. Lefse analysis between the CO, HFD and FLA-fed groups.

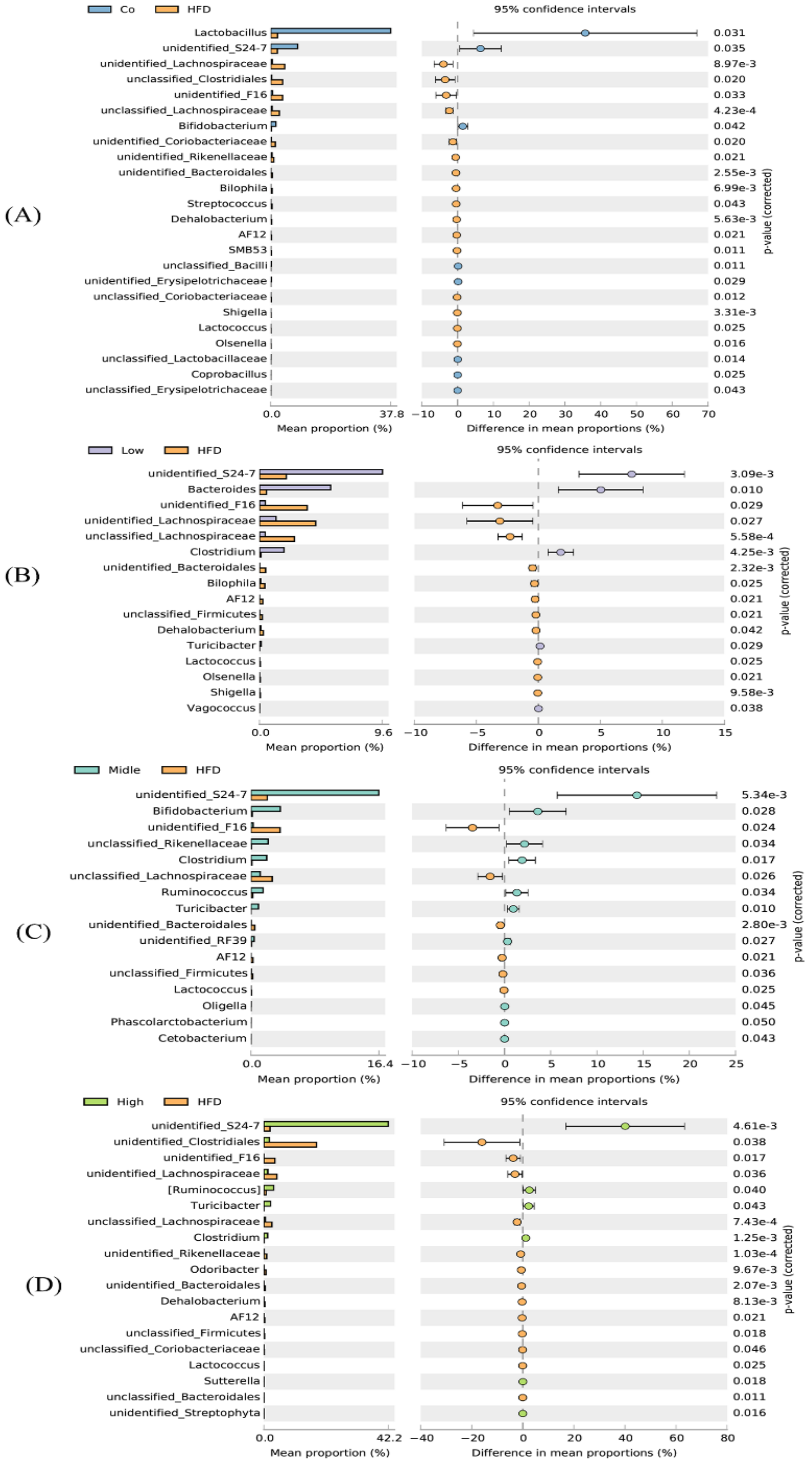

(A)
Co
HFD
95% confidence intervals
Lactobacillus
unidentified_S24-7
unidentified_Lachnospiraceae
unclassified_Clostridiales
unidentified_F16
unclassified_Lachnospiraceae
Bifidobacterium
unidentified_Coriobacteriaceae
unidentified_Rikenellaceae
unidentified_Bacteroidales
Bilophila
Streptococcus
Dehalobacterium
AF12
SMB53
unclassified_Bacilli
unidentified_Erysipelotrichaceae
unclassified_Coriobacteriaceae
Shigella
Lactococcus
Olsenella
unclassified_Lactobacillaceae
Coprobacillus
unclassified_Erysipelotrichaceae
0.031
0.035
8.97e-3
0.020
0.033
4.23e-4
0.042
0.020
0.021
2.55e-3
6.99e-3
0.043
5.63e-3
0.021
0.011
0.011
0.029
0.012
3.31e-3
0.025
0.016
0.014
0.025
0.043
0.0
37.8
Mean proportion (%)
−10 0 10 20 30 40 50 60 70
Difference in mean proportions (%)
p-value (corrected)
(B)
Low
HFD
95% confidence intervals
unidentified_S24-7
Bacteroides
unidentified_F16
unidentified_Lachnospiraceae
unclassified_Lachnospiraceae
Clostridium
unidentified_Bacteroidales
Bilophila
AF12
unclassified_Firmicutes
Dehalobacterium
Turicibacter
Lactococcus
Olsenella
Shigella
Vagococcus
3.09e-3
0.010
0.029
0.027
5.58e-4
4.25e-3
2.32e-3
0.025
0.021
0.021
0.042
0.029
0.025
0.021
9.58e-3
0.038
0.0
9.6
Mean proportion (%)
−10 −5 0 5 10 15
Difference in mean proportions (%)
p-value (corrected)
(C)
Midle
HFD
95% confidence intervals
unidentified_S24-7
Bifidobacterium
unidentified_F16
unclassified_Rikenellaceae
Clostridium
unclassified_Lachnospiraceae
Ruminococcus
Turicibacter
unidentified_Bacteroidales
unidentified_RF39
AF12
unclassified_Firmicutes
Lactococcus
Oligella
Phascolarctobacterium
Cetobacterium
5.34e-3
0.028
0.024
0.034
0.017
0.026
0.034
0.010
2.80e-3
0.027
0.021
0.036
0.025
0.045
0.050
0.043
0.0
16.4
Mean proportion (%)
−10 −5 0 5 10 15 20 25
Difference in mean proportions (%)
p-value (corrected)
(D)
High
HFD
95% confidence intervals
unidentified_S24-7
unidentified_Clostridiales
unidentified_F16
unidentified_Lachnospiraceae
[Ruminococcus]
Turicibacter
unclassified_Lachnospiraceae
Clostridium
unidentified_Rikenellaceae
Odoribacter
unidentified_Bacteroidales
Dehalobacterium
AF12
unclassified_Firmicutes
unclassified_Coriobacteriaceae
Lactococcus
Sutterella
unclassified_Bacteroidales
unidentified_Streptophyta
4.61e-3
0.038
0.017
0.036
0.040
0.043
7.43e-4
1.25e-3
1.03e-4
9.67e-3
2.07e-3
8.13e-3
0.021
0.018
0.046
0.025
0.018
0.011
0.016
0.0
42.2
Mean proportion (%)
−40 −20 0 20 40 60 80
Difference in mean proportions (%)
p-value (corrected)

Figure S5. Different bacteria among different dietary groups at five level, (A) CO vs. HFD; (B) Low vs. HFD; (C) Middle vs. HFD; (D) High vs. HFD. Low, 10% flaxseed group; Middle, 20% flaxseed group, High, 30% flaxseed group.

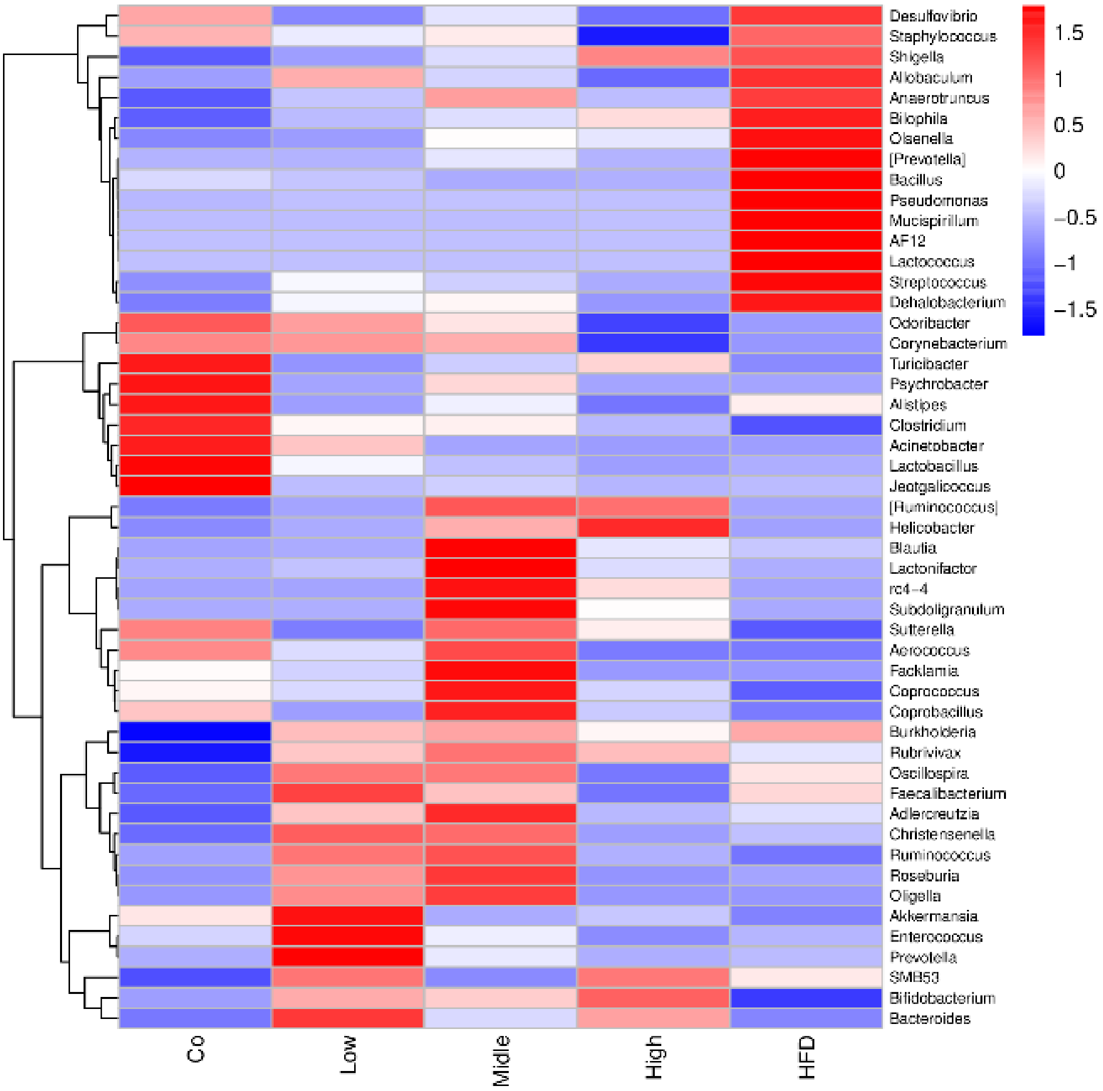

Figure S6. Heatmap analysis of the top abundance of gut microbe at genus level. Co, control group; HFD, high-fat diet; Low, 10% flaxseed group; Middle, 20% flaxseed group, High, 30% flaxseed group.

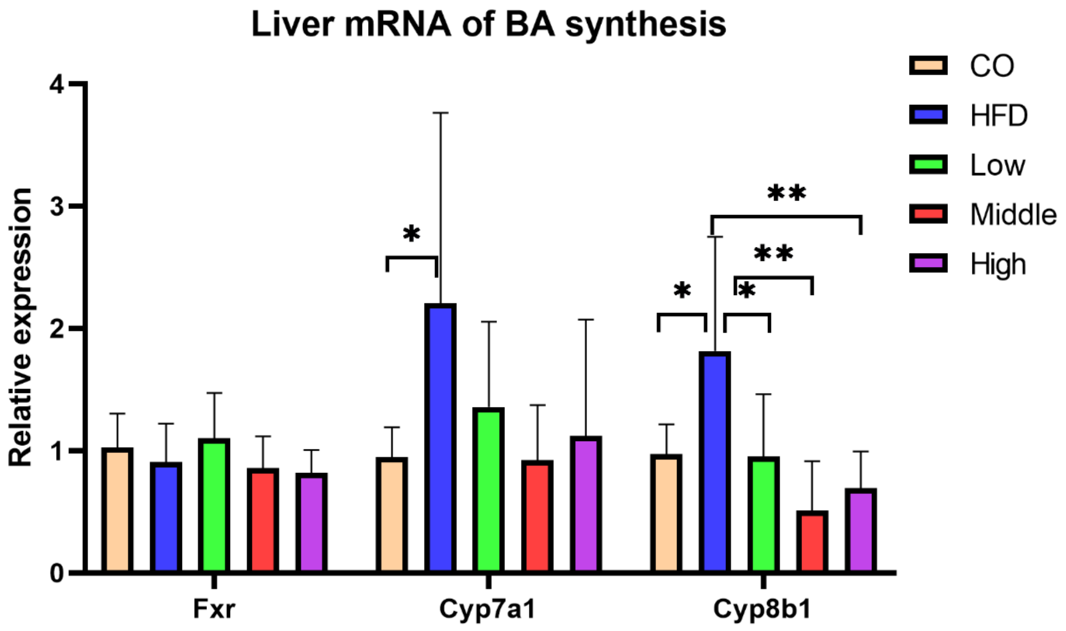

Figure S7. mRNA analysis of liver tissue to evaluate the expression of enzymes responsible for BA synthesis, including Fxr, Cyp7a1 and Cyp8b1. The relative mRNA levels of Fxr, Cyp7a1 and Cyp8b1 for BA synthesis in liver among the five groups. The data are presented as the mean ± SD. **p<0•01 (unpaired Student's t-test). n = 8 per group.

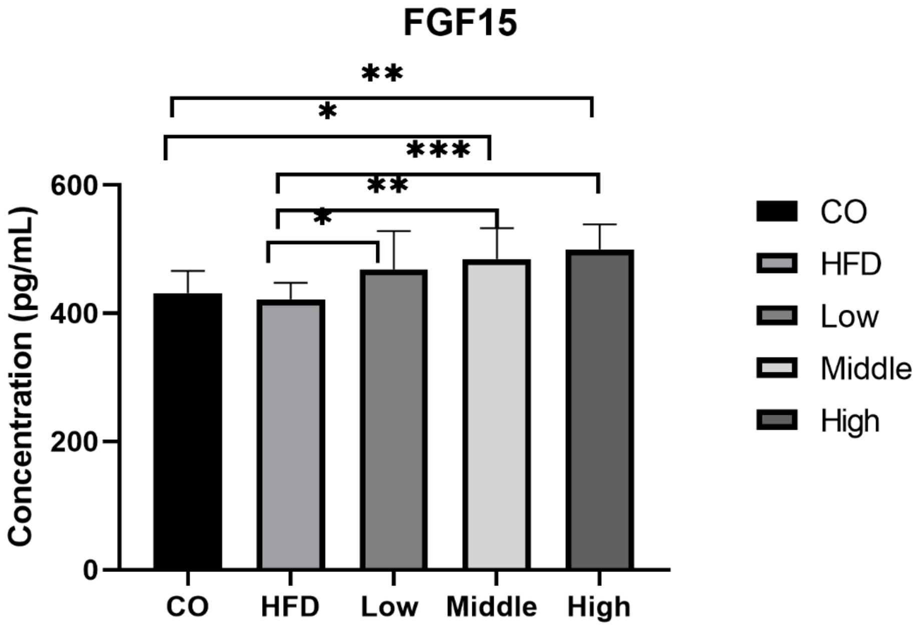

Figure S8. Differences in the plasma FGF15 concentration in five diet group. Serum FGF15 levels were detected by ELISA. Data are presented as the mean ± SD. **p<0•01 (unpaired Student's t-test).

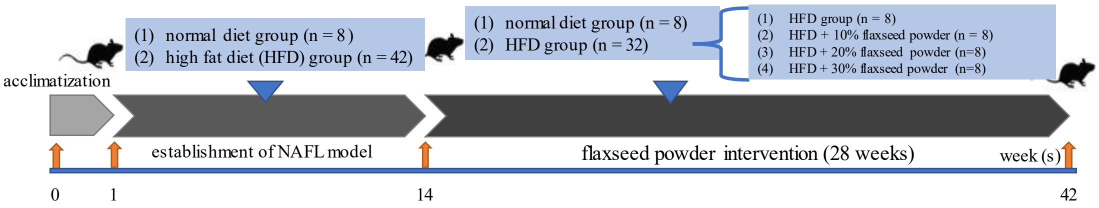

Figure S9. A timeline showing the experimental design.
