# Supplementary material for: Flaxseed Powder Attenuates Non-Alcoholic Steatohepatitis via Modulation of Gut Microbiota and Bile Acid Metabolism through Gut–Liver Axis
Source: Int J Mol Sci. 2021 Oct 8;22(19):10858. doi: 10.3390/ijms221910858 (PMC8509295; doi:10.3390/ijms221910858)
Supplement: Supplementary file 1 [file ijms-22-10858-s001.zip › Supplementary Tables.pdf]

Table S1. Fecal BA concentrations in the control and HFD group.

BAs (μg/g)	Control	HFD	P-value	HFD/Co	
	Mean ± SD	mean ± SD		Fold Change	log2(FC_HFD/Co)
alloLCA	2.09±1.60	1.46±0.77	0.64		0.52
LCA	35.87±24.57	106.41±37.62	0.00*	2.97	1.57
isoLCA	2.22±1.34	3.73±1.59	0.04*	1.68	0.75
NorDCA	0.50±0.52	0.51±0.25	0.79	1.02	0.03
12-ketoLCA	54.63±9.51	119.58±58.05	0.01*	2.19	1.13
7-ketoLCA	1.12±0.84	12.16±7.97	0.00*	10.88	3.44
beta-UDCA	2.72±1.98	9.43±4.45	0.00*	3.47	1.80
DCA	265.65±59.37	426.23±80.22	0.00*	1.60	0.68
CDCA	12.86±10.77	54.19±27.43	0.00*	4.21	2.08
UDCA	9.29±4.32	15.93±6.34	0.05	1.72	0.78
HDCA	68.31±39.39	159.77±54.25	0.01*	2.34	1.23
NorCA	2.14±2.20	0.03±0.07	0.00*	0.01	6.10
7,12-diketoLCA	1.64±1.85	0.16±0.46	0.05	0.10	3.33
alpha-MCA	48.17±25.40	83.65±69.29	0.43	1.74	0.80
UCA	4.70±5.81	4.86±3.43	0.49	1.03	0.05
beta-MCA	101.57±52.19	128.60±103.41	0.96	1.27	0.34
CA	70.59±103.39	117.90±170.65	0.37	1.67	0.74
ACA	12.84±15.39	13.36±17.66	0.96	1.04	0.06
beta-CA	141.28±42.92	203.29±80.85	0.10	1.44	0.52
GLCA	0.00±0.00	0.03±0.03	0.00*	60.50	5.92
GHDCA	0.00±0.01	0.04±0.06	0.04*	18.13	4.18
GUDCA	0.00±0.01	0.04±0.06	0.16	10.73	3.42
GDCA	0.70±0.71	1.74±1.29	0.05	2.48	1.31
LCA-3S	0.03±0.03	0.02±0.05	0.60	0.85	0.23
GCA	0.21±0.48	0.45±0.89	0.24	2.13	1.09
TLCA	0.02±0.03	0.46±0.47	0.01*	23.13	4.53
THDCA+TUDCA	0.77±1.31	3.14±6.29	0.02*	4.06	2.02
TDCA	1.67±1.97	8.35±10.28	0.03*	4.99	2.32
TCDCA	0.22±0.33	1.32±2.31	0.04*	5.95	2.57
TCA	6.04±10.97	18.65±35.07	0.05*	3.09	1.63
T-alpha-MCA	5.45±9.92	10.31±25.98	0.27	1.89	0.92
T-beta-MCA	5.37±9.81	10.46±26.41	0.27	1.95	0.96

The data are presented as the mean ± SD. BA species include unconjugated, taurine-conjugated, and glycine-conjugated BAs. The p value was determined by Mann–Whitney U test.

Table S2. Fecal BA concentrations in the FLA-fed and HFD group.

BAs (μg/g)	HFD	Low	Middle	High	Low/HFD		Middle/HFD		High/HFD	
	mean ± SD	mean ± SD	mean ± SD	mean ± SD	FC	p-value	FC	p-value	FC	p-value
alloLCA	1.46±0.77	3.04±1.24	3.01±1.38	5.69±3.51	2.08	0.02*	2.06	0.02*	3.90	0.00*
LCA	106.41±37.62	91.87±41.69	135.76±67.34	229.94±32.77	0.86	0.37	1.28	0.71	2.16	0.00*
isoLCA	3.73±1.59	4.92±1.76	7.48±5.35	13.66±4.79	1.32	0.27	2.01	0.23	3.66	0.00*
NorDCA	0.51±0.25	0.42±0.18	0.90±0.45	0.85±0.19	0.82	0.96	1.76	0.05	1.66	0.01*
12-ketoLCA	119.58±58.05	94.79±43.76	156.40±68.78	148.11±58.78	0.79	0.64	1.31	0.43	1.24	0.49
7-ketoLCA	12.16±7.97	5.23±3.68	8.41±6.08	13.00±4.00	0.43	0.08	0.69	0.27	1.07	0.64
beta-UDCA	9.43±4.45	14.98±6.24	17.79±10.87	25.27±3.72	1.59	0.05	1.89	0.02*	2.68	0.00*
DCA	426.23±80.22	445.61±76.05	525.08±70.87	514.60±43.06	1.05	0.49	1.23	0.04*	1.21	0.03*
CDCA	54.19±27.43	45.05±26.23	73.48±19.75	61.73±17.46	0.83	0.43	1.36	0.10	1.14	0.23
UDCA	15.93±6.34	25.66±10.14	29.52±12.18	36.56±9.56	1.61	0.07	1.85	0.02*	2.29	0.00*
HDCA	159.77±54.25	206.37±91.75	312.83±117.68	343.92±92.54	1.29	0.32	1.96	0.01*	2.15	0.00*
NorCA	0.03±0.07			0.20±0.15					6.41	0.02*
7,12-diketoLCA	0.16±0.46	1.12±1.25	0.67±0.84	0.76±0.76	6.83	0.07	4.13	0.09	4.65	0.09
alpha-MCA	83.65±69.29	101.66±60.92	180.64±155.05	383.15±134.62	1.22	0.43	2.16	0.05	4.58	0.00*
UCA	4.86±3.43	6.26±3.33	9.80±6.35	9.24±7.16	1.29	0.32	2.02	0.03*	1.90	0.23
beta-MCA	128.60±103.41	147.27±86.34	193.76±54.07	376.35±165.01	1.15	0.43	1.51	0.08	2.93	0.01*
CA	117.90±170.65	142.08±150.00	104.41±56.69	185.17±145.48	1.21	0.43	0.89	0.19	1.57	0.19
ACA	13.36±17.66	11.54±10.59	12.16±7.18	18.51±14.20	0.86	0.64	0.91	0.43	1.39	0.23
beta-CA	203.29±80.85	179.27±71.92	354.87±340.40	517.02±135.75	0.88	0.87	1.75	0.37	2.54	0.00*
GLCA	0.03±0.03	0.01±0.01	0.02±0.04	0.06±0.05	0.24	0.12	0.59	0.17	1.88	0.29
GHDCA	0.04±0.06	0.02±0.03	0.05±0.14	0.15±0.14	0.49	0.67	1.21	0.58	3.32	0.03*
GUDCA	0.04±0.06	0.02±0.02	0.06±0.14	0.14±0.13	0.51	0.75	1.30	0.83	3.12	0.06

GDCA	1.74±1.29	1.32±0.85	1.41±0.88	1.92±1.00	0.76	0.71	0.81	0.71	1.10	0.71
GCA	0.45±0.89	0.22±0.30	0.07±0.08	0.34±0.46	0.49	0.67	0.15	0.40	0.75	0.75
TLCA	0.46±0.47	0.14±0.18	0.13±0.09	0.64±0.43	0.31	0.06	0.28	0.04*	1.38	0.19
THDCA+TUDCA	3.14±6.29	1.28±0.81	1.41±0.78	4.17±2.06	0.41	0.71	0.45	0.49	1.33	0.02*
TDCA	8.35±10.28	3.53±2.68	3.99±1.95	8.01±4.68	0.42	0.37	0.48	0.43	0.96	0.56
TCDCA	1.32±2.31	0.30±0.18	0.42±0.29	0.89±0.48	0.23	0.16	0.32	0.34	0.67	0.32
TCA	18.65±35.07	6.66±4.32	5.15±1.63	7.71±6.30	0.36	0.87	0.28	0.23	0.41	0.64
T-alpha-MCA	10.31±25.98	1.95±2.03	1.22±0.95	9.41±12.12	0.19	0.63	0.12	0.87	0.91	0.41
T-beta-MCA	10.46±26.41	1.93±2.02	1.23±0.93	9.64±12.55	0.18	0.63	0.12	0.87	0.92	0.41

FC, Fold Change; 12-OH bile acids include CA, DCA and their taurine-conjugated and glycine-conjugated forms, ACA, and 23-norDCA; all other BAs are the non-12-OH bile acids. The data are presented as the mean ± SD. BA species include unconjugated, taurine-conjugated, and glycine-conjugated BAs. The p value was determined by Mann–Whitney U test.

Table S3. The number of microbial taxa at each level

Group	Sample	Phylum	Class	Order	Family	Genus	Species
**CO**	CO2_1	9	15	18	27	30	20
	CO2_2	7	13	16	24	28	18
	CO2_3	9	15	16	28	28	14
	CO2_4	8	14	20	34	44	32
	CO2_5	10	16	16	26	24	18
	CO2_6	8	16	19	33	37	25
	CO2_7	7	12	14	24	21	12
	CO2_12	8	13	16	29	25	18
**Low**	FLA1_2	7	13	17	29	35	17
	FLA1_4	6	12	19	33	37	25
	FLA1_5	6	12	17	32	38	24
	FLA1_6	7	13	18	31	35	24
	FLA1_10	8	15	24	47	54	30
	FLA1_14	8	17	27	40	50	31
	FLA1_15	9	16	20	37	51	25
	FLA1_16	7	14	19	38	47	19
**Middle**	FLA2_1	10	17	25	41	56	44
	FLA2_2	7	13	16	31	36	23
	FLA2_4	7	16	21	43	54	36
	FLA2_6	9	16	22	37	38	25
	FLA2_7	10	20	31	52	61	36
	FLA2_8	8	15	21	38	39	29
	FLA2_9	6	14	18	39	49	27
	FLA2_12	7	14	17	28	41	24
**High**	FLA3_1	7	14	24	34	40	23
	FLA3_3	8	15	19	30	28	16
	FLA3_4	6	13	17	27	31	18
	FLA3_8	8	16	20	33	35	18
	FLA3_12	9	16	25	36	42	20
	FLA3_13	6	15	17	25	27	17
	FLA3_14	8	16	25	39	43	23
	FLA3_15	9	16	20	33	41	26
HFD	HF2_1	7	13	19	37	42	23
	HF2_2	15	34	42	61	62	35
	HF2_3	7	15	19	30	35	19
	HF2_4	16	27	39	54	65	25
	HF2_5	9	14	15	23	28	22
	HF2_6	9	16	22	44	43	23
	HF2_7	7	13	20	35	37	16
	HF2_8	6	13	19	32	34	19

Co, control group; HFD, high-fat diet; Low, 10% flaxseed group; Middle, 20% flaxseed group, High, 30% flaxseed group.

Table S4. The ingredients of the flaxseed powder

Items	/100g	NRV%
**Energy**	2208 kJ	26%
**Protein**	20.0 g	33%
**Fat**	43.0 g	72%
Saturated fatty acids	4.0 g	20%
Trans fatty acids	0.0 g	
Monounsaturated fatty acids	9.0 g	
polyunsaturated fatty acids	30.0 g	
**Carbohydrate**	29.0 g	10%
**Dietary fiber**	24.0 g	96%
**Sodium**	70 mg	4%

NRV, nutrient reference values

Table S5. Compositions of experimental diets

**Ingredient**	**D12492**		**Chow**		**Low**		**Middle**		**High**	
Product	gm%	Kcal%	gm%	Kcal%	gm%	Kcal%	gm%	Kcal%	gm%	Kcal%
Protein	26.2	20	19.3	20	26.2	20	26.2	20	26.2	20
Carbohydrate	26.3	20	67.6	70	26.3	20	26.3	20	26.3	20
Fat	34.9	60	2.4	10	34.9	60	34.9	60	34.9	60
Ingredient	gm	kcal	gm	kcal	gm	kcal	gm	kcal	gm	kcal
Casein, 80 Mesh	200	800	200	800	184.5	738	169	676	153.6	614.4
L-Cystine	3	12	3	12	3	12	3	12	3	12
Flaxseed (protein)	0	0	0	0	15.5	62	31	124	46.4	185.6
Flaxseed (fat)	0	0	0	0	33.3	299.7	66.6	599.4	99.8	898.2
Flaxseed (carbohydrate)	0	0	0	0	3.9	15.6	7.7	30.8	11.6	46.4
Flaxseed (dietary fiber)	0	0	0	0	18.6	0	37.2	0	55.6	0
Corn Starch	0	0	452.2	1809	0	0	0	0	0	0
Maltodextrin 10	125	500	75	300	121.1	484.4	117.3	469.2	113.4	453.6
Sucrose	68.8	275.2	172.8	691	68.8	275.2	68.8	275.2	68.8	275.2
Cellulose, BW200	50	0	50	0	31.4	0	12.8	0	0	0
Soybean Oil	25	225	25	225	25	225	25	225	25	225

Lard	245	2205	0	0	211.7	1905.3	178.4	1605.6	145.2	1306.8
Mineral Mix S10026	10	0	10	0	10	0	10	0	10	0
DiCalcium Phosphate	13	0	13	0	13	0	13	0	13	0
Calcium Carbonate	5.5	0	5.5	0	5.5	0	5.5	0	5.5	0
Potassium Citrate, 1 H2O	16.5	0	16.5	0	16.5	0	16.5	0	16.5	0
Vitamin Mix V10001	10	40	10	40	10	40	10	40	10	40
Choline Bitartrate	2	0	2	0	2	0	2	0	2	0
FD&C Blue Dye #1	0.05	0	0	0	0.05	0	0.05	0	0.05	0
Total	773.8	4057.2	1035	3850	792.4	4057.2	811	4057.2	829.4	4057.2

Low, 10% flaxseed powder; Middle, 20% flaxseed powder; High, 30% flaxseed powder

Table S6. The sequences of primers of the C57BL/6J mouse used in quantitative real-time polymerase chain reaction (qRT-PCR)

**GENE**	**Forward primer (5'->3')**	**Reverse primer (5'->3')**
Fxr	TTCGAAAGAGCGGCATCTCC	TAGGACATCGAGCAGAGGCT
Cyp8b1	GGACAGCCTATCCTTGGTGA	CGGAACTTCCTGAACAGCTC
Cyp7a1	GTACCTTGATGAAAGTGGGAA	ATTGCTTGATTTCTTGGACAG
Fgfr4	TGTCAAATTCCGCTGTCCAG	ACCACACTTTCCATCACCAG
GAPDH	AAGAAGGTGGTGAAGCAGG	GAAGGTGGAAGAGTGGGAGT

Fxr, farnesoid X receptor, Cyp7a1, cholesterol 7α-hydroxylase; Cyp8b1, sterol 12α-hydroxylase; Fgfr4, fibroblast growth factor receptor 4;
